# Supplementary material for: A Comprehensive Assessment of Ultraviolet-Radiation-Induced Mutations in Flammulina filiformis Using Whole-Genome Resequencing
Source: J Fungi (Basel). 2024 Mar 20;10(3):228. doi: 10.3390/jof10030228 (PMC10971301; doi:10.3390/jof10030228)
Supplement: Supplementary file 1 [file jof-10-00228-s001.zip › Supplementary Material S8/KEGG annotation/out/64381550635650.os/KO/out_map/map04360.html]

KEGG PATHWAY: Axon guidance - Reference pathway


|  |  |
| --- | --- |
| **Axon guidance - Reference pathway** |  |

[
Pathway menu
| Organism menu
| Pathway entry
| Show description
| User data mapping
]

Axon guidance represents a key stage in the formation of neuronal network. Axons are guided by a variety of guidance factors, such as netrins, ephrins, Slits, and semaphorins. These guidance cues are read by growth cone receptors, and signal transduction pathways downstream of these receptors converge onto the Rho GTPases to elicit changes in cytoskeletal organization that determine which way the growth cone will turn.

|  |  |
| --- | --- |
| Reference pathway | 100% |
